# Secure Authentication Protocol for Wireless Sensor Networks in Vehicular Communications

**DOI:** 10.3390/s18103191

**Published:** 2018-09-21

**Authors:** SungJin Yu, JoonYoung Lee, KyungKeun Lee, KiSung Park, YoungHo Park

**Affiliations:** 1 School of Electronics Engineering, Kyungpook National University, Daegu 41566, Korea; darkskiln@naver.com (S.Y.); harry250@naver.com (J.L.); 2 Samsung Electronics, Suwon 16677, Korea; crypto.knu@gmail.com

**Keywords:** authentication, wireless sensor network, vehicular communications, formal security analysis, BAN logic, AVISPA

## Abstract

With wireless sensor networks (WSNs), a driver can access various useful information for convenient driving, such as traffic congestion, emergence, vehicle accidents, and speed. However, a driver and traffic manager can be vulnerable to various attacks because such information is transmitted through a public channel. Therefore, secure mutual authentication has become an important security issue, and many authentication schemes have been proposed. In 2017, Mohit et al. proposed an authentication protocol for WSNs in vehicular communications to ensure secure mutual authentication. However, their scheme cannot resist various attacks such as impersonation and trace attacks, and their scheme cannot provide secure mutual authentication, session key security, and anonymity. In this paper, we propose a secure authentication protocol for WSNs in vehicular communications to resolve the security weaknesses of Mohit et al.’s scheme. Our authentication protocol prevents various attacks and achieves secure mutual authentication and anonymity by using dynamic parameters that are changed every session. We prove that our protocol provides secure mutual authentication by using the Burrows–Abadi–Needham logic, which is a widely accepted formal security analysis. We perform a formal security verification by using the well-known Automated Validation of Internet Security Protocols and Applications tool, which shows that the proposed protocol is safe against replay and man-in-the-middle attacks. We compare the performance and security properties of our protocol with other related schemes. Overall, the proposed protocol provides better security features and a comparable computation cost. Therefore, the proposed protocol can be applied to practical WSNs-based vehicular communications.

## 1. Introduction

Wireless sensor networks (WSNs), in conjunction with intelligent transport systems (ITS) and embedded technology, have advanced to such an extent that drivers can make full use of various information such as traffic congestion, vehicle accidents, and speed. To provide these useful services, a sensor in the vehicle collects data on the vehicle and surrounding area and sends it to the traffic manager through a sink node. The traffic manager in the traffic management office receives data from vehicle sensors and can monitor a vehicle and the surrounding area to provide useful data to the driver in real time. However, a malicious adversary can easily obtain and modify the data because it is transmitted via a public network. Therefore, the authentication protocol between the vehicle and user in vehicular communications has become a very important security issue. In the last few decades, numerous authentication schemes for WSNs have been proposed to ensure secure communications and user privacy [1,2,3,4,5,6,7,8]. In 2006, Wong et al. [9] proposed a dynamic ID-based user authentication scheme for WSNs. However, Das et al. [10] showed that Wong et al.’s [9] scheme is vulnerable to the stolen verifier attack and proposed an improved two-factor authentication scheme to overcome these security problems. In 2010, Chen et al. [11] demonstrated that Das et al.’s scheme [10] cannot provide secure mutual authentication and cannot resist parallel session attacks. To resolve this problem, they proposed a robust mutual authentication scheme for WSNs. Khan et al. [12] also showed that Das et al.’s scheme [10] cannot prevent the privileged insider and bypassing attacks, nor can it provide mutual authentication and the password changing phase. To overcome these security weaknesses, they proposed a two-factor user authentication protocol that uses secret parameters. In 2011, Yeh et al. [13] found that Das et al.’s scheme cannot resist the insider attack and provide mutual authentication, which are essential security requirements for the WSNs. They proposed a secured authentication protocol for WSNs that uses elliptic curve cryptography (ECC). Unfortunately, Han [14] pointed out that Yeh et al.’s scheme cannot provide mutual authentication, perfect forward secrecy, and key agreement. To resolve the security weaknesses of Yeh et al.’s scheme, Shi et al. [15] proposed a new user authentication protocol for WSNs using ECC. However, Choi et al. [16] showed that Shi et al.’s [15] scheme is vulnerable to a smartcard being stolen, sensor energy exhaustion, and session key attacks. They proposed a new user authentication protocol based on ECC.

In the last few decades, numerous protocols for secure vehicle communications have been proposed [17,18,19,20,21,22,23,24,25]. In 2008, Zhang et al. [17] proposed an efficient roadside unit (RSU)-aided message authentication scheme that uses a hash message authentication code (HMAC) for vehicular communications networks. Zhang et al. also proposed [18] an efficient message authentication scheme for vehicular communications. Lu et al. [19] proposed an efficient conditional privacy preservation protocol for secure vehicular communications that uses bilinear pairing. However, their protocol is not efficient in resource-constrained vehicular ad hoc networks (VANETs) because it has used multiple anonymous key and has high latency for generating of pseudo-random keys [20]. In 2014, Chuang and Lee [21] proposed an authentication mechanism for vehicle to vehicle communications in VANETs. However, in 2016, Kumari et al. [22] showed that Chuang and Lee’s authentication protocol is vulnerable to insider and impersonation attacks, and they proposed an enhanced authentication protocol for VANETs. In 2017, Mohit et al. [23] also proposed an authentication protocol for WSNs in vehicle communications. Mohit et al. claimed that their proposed scheme can resist various attacks such as smartcard stolen, impersonation, and untraceable attacks. In this paper, however, we demonstrate that their scheme cannot resist impersonation and trace attacks. In addition, we show that Mohit et al.’s scheme cannot provide anonymity, session key security, and mutual authentication. We propose a secure authentication protocol for WSNs in vehicle communications that overcomes these security weaknesses.

### 1.1. Threat Model

To analyze the security of our proposed scheme, we introduce the Dolev–Yao (DY) threat model, which is widely used to evaluate the security of a protocol. The detailed assumptions of the DY threat model are as follows:

- An adversary can modify, eavesdrop, insert or delete the transmitted messages over a public channel.
- An adversary can obtain a lost or smartcard stolen, and he/she can also extract the information stored in the smartcard [26,27].
- An adversary can perform various attacks such as impersonation, trace, smartcard stolen, and replay attacks.

### 1.2. Our Contributions

The main contributions of this paper are as follows:

- We demonstrate that Mohit et al.’s scheme is vulnerable to various attacks such as impersonation and trace attacks. In addition, we point out that their scheme cannot provide mutual authentication, session key security and anonymity.
- We propose a secure authentication protocol for WSNs in vehicular communications to resolve these security weaknesses. Our proposed protocol prevents impersonation and trace attacks, and also achieves anonymity, session key security and secure mutual authentication. In addition, the proposed scheme is efficient because it utilizes only hash function and XOR operation in authentication phase.
- We prove that our protocol provides secure mutual authentication by using the broadly accepted Burrows–Abadi–Needham (BAN) logic [28]. We also perform an informal analysis to demonstrate the security of the proposed protocol against various attacks such as impersonation and trace attacks.
- We compare the performance of our scheme against those of related existing schemes and perform a formal security verification by using the widespread Automated Validation of Internet Security Protocols and Applications (AVISPA) simulation software tool.

### 1.3. Paper Outline

The remainder of this paper is organized as follows. In Section 2, we introduce the vehicular communications system model. In Section 3 and Section 4, we review Mohit et al.’s authentication scheme and analyze its security weaknesses. In Section 5, we propose a secure authentication protocol for WSNs in vehicular communications to resolve the security problems of their scheme. In Section 6, we present an informal analysis on the security of our protocol and prove that it achieves secure mutual authentication by using BAN logic. In Section 7 and Section 8, we present the formal security verification with the AVISPA simulation tool and compare the performance of our protocol with that of related protocols. Finally, we present our conclusions in Section 9.

## 2. System Model

**Figure 1:**
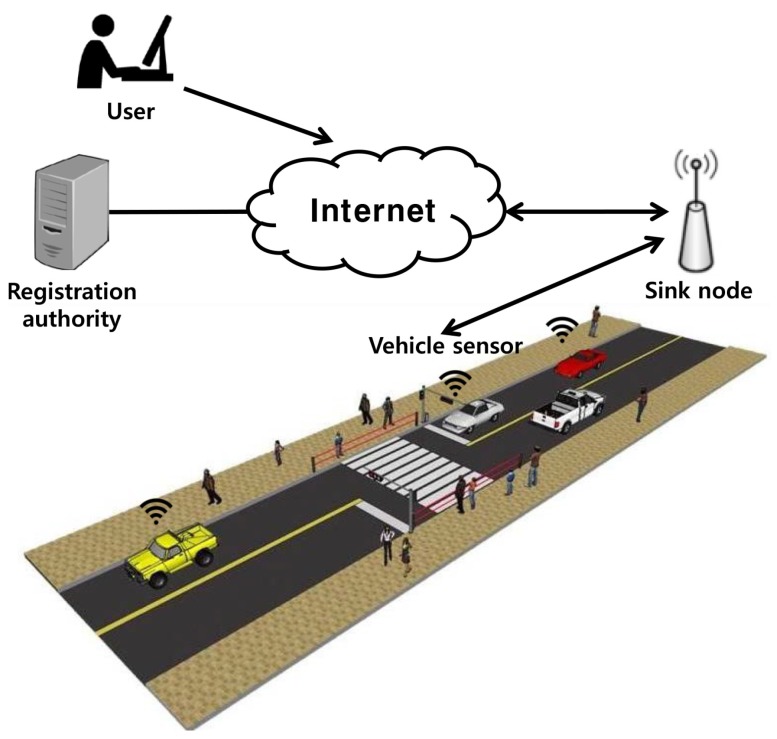
Vehicular communications system model.

In this section, we introduce a vehicular communication system using WSNs and essential security requirements. There are three entities involved in the vehicular communications system: the vehicle sensor, sink node, and user. The vehicular communications system model is shown in Figure 1.

The vehicular communications system consists of two parts: the WSNs and vehicle and the user and sink node. The vehicle sensor is deployed in the vehicle and collects data on the traffic and surrounding area in real time, which it then sends to the sink node. After receiving the data from the vehicle sensor, the sink node stores it for the user. The user can control the response to traffic jams, speed, and emergency situations based on the data collected by the sink node.

The numerous authentication protocols [29,30,31] have defined security requirements in order to explain their security goals. Therefore, we also define the essential security requirements to explain and ensure our security goals.

- **Untraceability and anonymity.** In a modern vehicular communication system, user’s real identity and location data are very sensitive information. For these reason, an adversary cannot trace a user’s location and know the user’s real identity to guarantee a privacy of user.
- **Secure mutual authentication.** A secure mutual authentication is known for a essential security requirement in VANETs in order to guarantee that only the legitimate users should access the services and communicate securely with each other [32].
- **Confidentiality.** In our system, the user, sink node, and vehicle sense can freely communicate among themselves through a internet. However, an adversary can try to obtain various pieces of information from users such as traffic congestion, speed, and vehicle accident because it is transmitted in a public channel. Therefore, a confidentiality must be guaranteed and the transmitted data is only known to legitimate user in order to ensure a security.

## 3. Review of Mohit et al.’s Scheme

In this section, we review Mohit et al.’s authentication protocol for WSNs, which consists of three phases: system setup, user registration, and user login and authentication. Table 1 presents the notations used in this paper.

### 3.1. System Setup Phase

When a driver wants to deploy a sensor in a vehicle, the registration authority (RA) registers the vehicle sensor in the network. In addition, RA stores various data on the vehicle such as the vehicle number, engine, battery, and insurance in a database.

### 3.2. User Registration Phase

**Figure 2:**
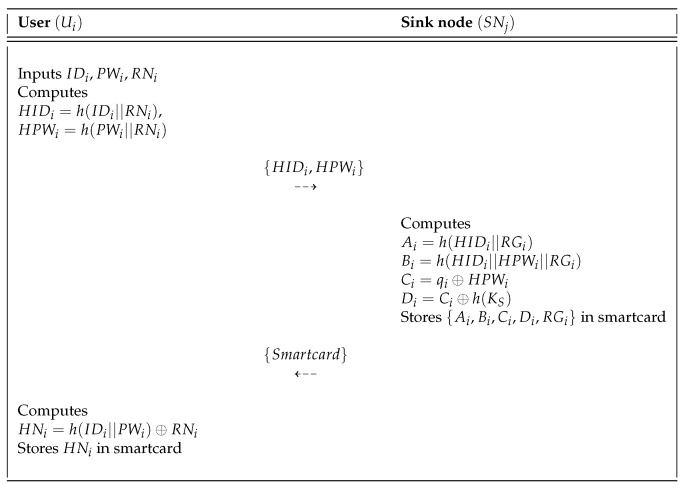
User registration phase of the Mohit et al.’s scheme.

If a new traffic manager $U_{i}$ wants to register him or herself, $U_{i}$ must send the registration request message to the sink node $SN_{j}$ first. The user registration phase of Mohit et al.’s scheme is shown in Figure 2, and the detailed steps are described as follows.

**Step** **1:** $U_{i}$ chooses an identity $ID_{i}$, password $PW_{i}$, and random nonce $RN_{i}$. $U_{i}$ then computes $HID_{i}=h(ID_{i}||RN_{i})$, $HPW_{i}=h(PW_{i}||RN_{i})$ and sends them to the sink node via a secure channel.
**Step** **2:** $SN_{j}$ selects a random nonce $RG_{i}$ and random number $q_{i}$, and then $SN_{j}$ computes $A_{i}=h(HID_{i}||RG_{i})$, $B_{i}=h(HID_{i}||HPW_{i}||RG_{i})$, $C_{i}=q_{i}⊕HPW_{i}$, and $D_{i}=C_{i}⊕h\left(K_{s}\right)$. After that, $SN_{j}$ stores $\left\{A_{i},B_{i},C_{i},D_{i},RG_{i}\right\}$ in the smartcard and issues the smartcard to $U_{i}$ through a secure channel.
**Step** **3:** Upon receiving the smartcard, $U_{i}$ computes $HN_{i}=h(ID_{i}||PW_{i})⊕RN_{i}$ and stores it in the smartcard. Ultimately, the smartcard contains $\left\{A_{i},B_{i},C_{i},D_{i},RG_{i},HN_{i}\right\}$.

### 3.3. User Login and Authentication Phase

**Figure 3:**
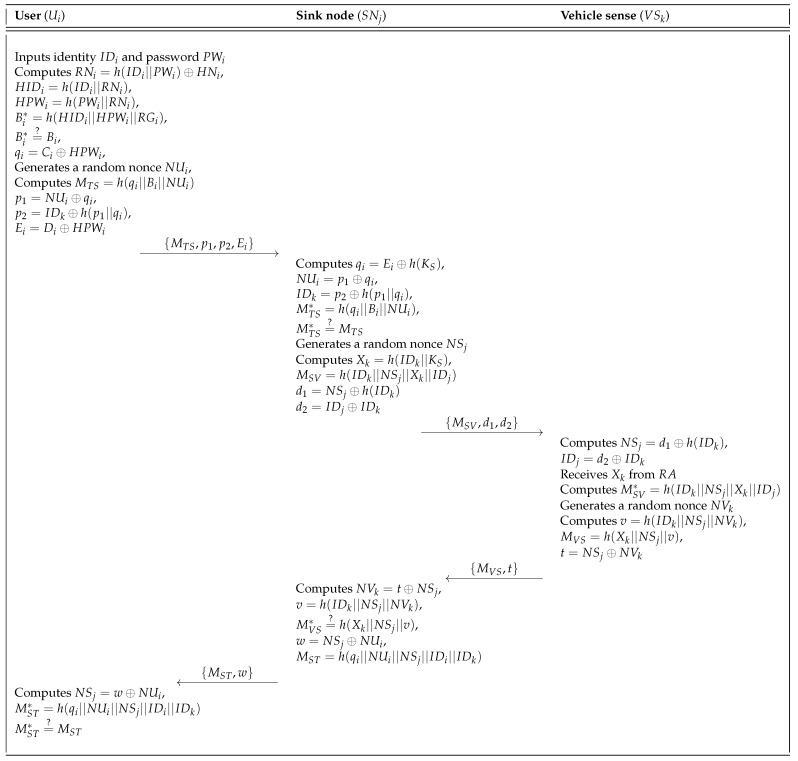
User login and authentication phase of the Mohit et al.’s scheme.

If a user $U_{i}$ wants to access the system, $U_{i}$ must send the login request message to the sink node $SN_{j}$. After receiving the login request message from $U_{i}$, $SN_{j}$ checks whether it is legitimate. If it is valid, $SN_{j}$ performs the authentication phase. The user login and authentication phase of Mohit et al.’s scheme is shown in Figure 3. The detailed steps of this phase are described as follows.

**Step** **1:** $U_{i}$ inserts the smartcard into a card reader and inputs $ID_{i}$ and $PW_{i}$. The smartcard then computes $RN_{i}=h(ID_{i}||PW_{i})⊕HN_{i}$, $HID_{i}=h(ID_{i}||RN_{i})$, $HPW_{i}=h(PW_{i}||RN_{i})$, and $B_{i}^{*}=h(HID_{i}||HPW_{i}||RG_{i})$. Then, the smartcard checks whether $B_{i}^{*}\overset{?}{=}B_{i}$. If it is equal, the smartcard computes $q_{i}=C_{i}⊕HPW_{i}$ and generates a random nonce $NU_{i}$. The smartcard also computes $M_{TS}=h(q_{i}\left|\right|B_{i}||NU_{i})$, $p_{1}=NU_{i}⊕q_{i}$, $p_{2}=ID_{k}⊕h(p_{1}\left|\right|q_{i})$ and $E_{i}=D_{i}⊕HPW_{i}$. Finally, the smartcard sends the login request message $\left\{M_{TS},p_{1},p_{2},E_{i}\right\}$ to $SN_{j}$ via a public channel.
**Step** **2:** After receiving the login request message from $U_{i}$, $SN_{j}$ retrieves $q_{i}=E_{i}⊕h\left(K_{s}\right),NU_{i}=p_{1}⊕q_{i}$ and $ID_{k}=p_{2}⊕h(p_{1}\left|\right|q_{i})$. Then, $SN_{j}$ computes $M_{TS}^{*}=h(q_{i}\left|\right|B_{i}||NU_{i})$ and checks whether $M_{TS}^{*}$ is equal to $M_{TS}$. Then, $SN_{j}$ generates a random nonce $NS_{j}$ and computes $X_{k}=h(ID_{k}\left|\right|K_{s})$, $M_{SV}=h(ID_{k}||NS_{j}\left|\right|X_{k}||ID_{j})$, $d_{1}=NS_{j}⊕h\left(ID_{k}\right),d_{2}=ID_{j}⊕ID_{k}$. Finally, $SN_{j}$ sends $\left\{M_{SV},d_{1},d_{2}\right\}$ to the vehicle sensor.
**Step** **3:** Upon receiving the message $\left\{M_{SV},d_{1},d_{2}\right\}$, the vehicle sensor $VS_{k}$ retrieves $NS_{j}=d_{1}⊕h\left(ID_{k}\right)$ and $ID_{j}=d_{2}⊕ID_{k}$. Then, $VS_{k}$ checks the freshness of $NS_{j}$. If it is fresh, $VS_{k}$ sends $ID_{k}$ and requests the sink node’s master key $X_{k}$ from RA. After receiving $X_{k}$ from RA through a secure channel, $VS_{k}$ computes $M_{SV}^{*}=h(ID_{k}||NS_{j}\left|\right|X_{k}||ID_{j})$ and checks whether $M_{SV}^{*}\overset{?}{=}M_{SV}$. If it is verified, $VS_{k}$ chooses a random nonce $NV_{k}$ and computes $v=h(ID_{k}||NS_{j}||NV_{k})$, $M_{VS}=h(X_{k}||NS_{j}\left||v\right)$, and $t=NS_{j}⊕NV_{k}$. Finally, $VS_{k}$ sends $\left\{M_{VS},t\right\}$ to $SN_{j}$.
**Step** **4:** After receiving the message $\left\{M_{VS},t\right\}$, $SN_{j}$ retrieves $NV_{k}=t⊕NS_{j}$ and computes $v=h(ID_{k}||NS_{j}||NV_{k}),M_{VS}^{*}=h(X_{k}||NS_{j}\left||v\right)$. Then, $SN_{j}$ checks whether $M_{VS}^{*}\overset{?}{=}M_{VS}$ is correct. If it is correct, $SN_{j}$ computes $w=NS_{j}⊕NU_{i}$, $M_{ST}=h(q_{i}||NU_{i}||NS_{j}||ID_{i}||ID_{k})$ and sends $\left\{M_{ST},w\right\}$ to $U_{i}$.
**Step** **5:** Upon receiving the message $\left\{M_{ST},w\right\}$ from $SN_{j}$, $U_{i}$ retrieves $NS_{j}=w⊕NU_{i}$ and computes $M_{ST}^{*}=h(q_{i}||NU_{i}||NS_{j}||ID_{i}||ID_{k})$, and then $U_{i}$ checks whether $M_{ST}^{*}\overset{?}{=}h(q_{i}||NU_{i}||NS_{j}||ID_{i}||ID_{k})$ is correct. If they are equal, mutual authentication has been successfully achieved.

### 3.4. Password Change Phase

**Figure 4:**
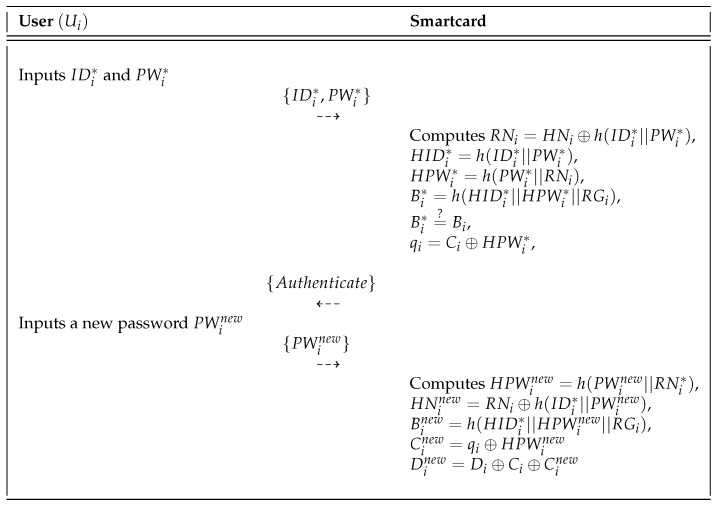
Password change phase of the Mohit et al.’s scheme.

$U_{i}$ can freely update his or her password when desired. The password change phase is described in Figure 4 and the detailed steps of this phase are as follows.

**Step** **1:** $U_{i}$ inserts smartcard in the card reader and inputs the identity $ID_{i}^{*}$ and password $PW_{i}^{*}$, and then $U_{i}$ submits $\left\{ID_{i}^{*},PW_{i}^{*}\right\}$ to the card reader via a secure channel.
**Step** **2:** After receiving $\left\{ID_{i}^{*},PW_{i}^{*}\right\}$, the smartcard computes $RN_{i}=HN_{i}⊕h(ID_{i}^{*}||PW_{i}^{*})$, $HID_{i}^{*}=h(ID_{i}^{*}||PW_{i}^{*})$, $HPW_{i}^{*}=h(PW_{i}^{*}||RN_{i})$, and $B_{i}^{*}=h(HID_{i}^{*}||HPW_{i}^{*}||RG_{i})$. It checks whether $B_{i}^{*}\overset{?}{=}B_{i}$. If this is verified, the smartcard sends the authentication message and requests a new password from $U_{i}$. After receiving the authentication message from smartcard, $U_{i}$ inputs the new password $PW_{i}^{new}$.
**Step** **3:** The smartcard calculates $HPW_{i}^{new}=h(PW_{i}^{new}||RN_{i}^{*})$, $HN_{i}^{new}=RN_{i}⊕h(ID_{i}^{*}||PW_{i}^{new})$, $B_{i}^{new}=h(HID_{i}^{*}||HPW_{i}^{new}||RG_{i})$, $C_{i}^{new}=q_{i}⊕HPW_{i}^{new}$, and $D_{i}^{new}=D_{i}⊕C_{i}⊕C_{i}^{new}$ by using the new password of $U_{i}$. Finally, smartcard replaces $\left\{HN_{i},B_{i},C_{i},D_{i}\right\}$ with $\left\{HN_{i}^{new},B_{i}^{new},C_{i}^{new},D_{i}^{new}\right\}$.

## 4. Cryptanalysis of Mohit et al.’s Scheme

In this section, we discuss the security weaknesses of Mohit et al.’s scheme. They asserted that their scheme is secure against trace and impersonation attack, and they showed that their scheme can provide anonymity, session key security and secure mutual authentication. However, here we demonstrate that Mohit et al.’s scheme does not resist the following attacks.

### 4.1. Impersonation Attack

If an adversary $U_{a}$ tries to impersonate a legitimate user, $U_{a}$ can successfully generate a login request message of legitimate user $\left\{M_{TS},p_{1},p_{2},E_{i}\right\}$. According to Section 1.1, we can assume that $U_{a}$ obtains the smartcard of the legitimate user $U_{i}$ and extracts the values $\left\{B_{i},C_{i},D_{i}\right\}$ stored in smartcard and that $U_{a}$ has the messages transmitted in the previous session. Here, we show that Mohit et al.’s scheme does not prevent an impersonation attack.

**Step** **1:** $U_{a}$ computes $HPW_{i}=D_{i}⊕E_{i}$, $q_{i}=C_{i}⊕HPW_{i}$, $NU_{i}=p_{1}⊕q_{i}$, $ID_{k}=p_{2}⊕h(p_{1}\left|\right|q_{i})$, and $M_{TS}=h(q_{i}\left|\right|B_{i}||NU_{i})$, where $E_{i}$, $p_{1}$, and $p_{2}$ are messages of the previous session.
**Step** **2:** $U_{a}$ can obtain the secret parameters $q_{i}$, $B_{i}$, and $HPW_{i}$ and a random nonce $NU_{i}$. $U_{a}$ then chooses a random nonce $RU_{a}$ and computes $M_{TSa}=h(q_{i}\left|\right|B_{i}||NU_{a})$, $p_{1a}=NU_{a}⊕q_{i}$, and $p_{2a}=ID_{k}⊕h(p_{1a}\left|\right|q_{i})$. Finally, $U_{a}$ generates the login request message $\left\{M_{TSa},p_{1a},p_{2a},E_{i}\right\}$ and sends it to the sink node $SN_{j}$.
**Step** **3:** After receiving the login request message from $U_{a}$, $SN_{j}$ retrieves $q_{i}=E_{i}⊕h\left(K_{s}\right),NU_{a}=p_{1a}⊕p2_{2a}$, and $ID_{k}=p_{2a}⊕h(p_{1a}\left|\right|q_{i})$. $SN_{j}$ then computes $M_{TS}^{*}=h(q_{i}\left|\right|B_{i}||NU_{a})$ and checks whether $M_{TS}^{*}$ is equal to $M_{TSa}$. Then, $SN_{j}$ generates a random nonce $NS_{j2}$ and computes $X_{k}=h(ID_{k}\left|\right|K_{s})$, $M_{SV2}=h(ID_{k}||NS_{j2}\left|\right|X_{k}||ID_{j})$, $d_{1}=NS_{j2}⊕h\left(ID_{k}\right)$, and $d_{2}=ID_{j2}⊕ID_{k}$. Finally, $SN_{j}$ sends $\left\{M_{SV2},d_{1},d_{2}\right\}$ to the vehicle sensor.
**Step** **4:** Upon receiving the message $\left\{M_{SV2},d_{1},d_{2}\right\}$, the vehicle sensor $VS_{k}$ retrieves $NS_{j2}=d_{1}⊕h\left(ID_{k}\right)$ and $ID_{j}=d_{2}⊕ID_{k}$, and then $VS_{k}$ checks the freshness of $NS_{j2}$. If it is fresh, $VS_{k}$ sends $ID_{k}$ and requests the sink node’s master key $X_{k}$ from RA. After receiving $X_{k}$ from RA through a secure channel, $VS_{k}$ computes $M_{SV2}^{*}=h(ID_{k}||NS_{j2}\left|\right|X_{k}||ID_{j})$ and checks whether $M_{SV2}^{*}\overset{?}{=}M_{SV2}$. If it is verified, $VS_{k}$ chooses a random nonce $NV_{k2}$ and computes $v=h(ID_{k}||NS_{j2}||NV_{k2})$, $M_{VS2}=h(X_{k}||NS_{j2}\left||v\right)$, and $t=NS_{j2}⊕NV_{k2}$. Finally, $VS_{k}$ sends $\left\{M_{VS2},t\right\}$ to $SN_{j}$.
**Step** **5:** After receiving the message $\left\{M_{VS2},t\right\}$, $SN_{j}$ retrieves $NV_{k2}=t⊕NS_{j2}$ and computes $v=h(ID_{k}||NS_{j2}||NV_{k2})$ and $M_{VS2}^{*}=h(X_{k}||NS_{j2}\left||v\right)$. Then, $SN_{j}$ checks whether $M_{VS2}^{*}\overset{?}{=}M_{SV2}$ is correct. If it is correct, $SN_{j}$ computes $w=NS_{j2}⊕NU_{a}$ and $M_{ST2}=h(q_{i}||NU_{a}||NS_{j2}||ID_{i}||ID_{k})$ and sends $\left\{M_{ST2},w\right\}$ to $U_{a}$.
**Step** **6:** Upon receiving the message $\left\{M_{ST2},w\right\}$ from $SN_{j}$, $U_{a}$ successfully achieves mutual authentication.

Therefore, Mohit et al.’s scheme is vulnerable to impersonation attacks.

### 4.2. Trace Attack and Anonymity Preservation

According to Section 4.1, an adversary $U_{a}$ can obtain the real identities of the vehicle sensor and sink node. First, $U_{a}$ retrieves the vehicle sensor’s real identity $ID_{k}=p_{2}⊕h(p_{1}\left|\right|q_{i})$ and then computes $NS_{j}=d_{1}⊕h\left(ID_{k}\right)$. Finally, $U_{a}$ retrieves the sink node’s real identity $ID_{j}=d_{2}⊕ID_{k}$. For this reason, Mohit et al.’s scheme does not prevent trace attack or provide anonymity.

### 4.3. Mutual Authentication

In Section 4.1, we demonstrate that Mohit et al.’s scheme does not resist impersonation attacks. An adversary $U_{a}$ can compute the login request message $\left\{M_{TS},p_{1},p_{2},E_{i}\right\}$ and successfully achieve mutual authentication with $VS_{k}$. In addition, the sink node $SN_{j}$ cannot compute the authentication message $M_{ST}=h(q_{i}||NU_{i}||NS_{j}||ID_{i}||ID_{k})$ in the login and authentication phase because $SN_{j}$ does not know the real identity of $U_{i}$. Therefore, Mohit et al.’s scheme does not provide secure mutual authentication.

### 4.4. Session Key Security

Mohit et al. claimed that their scheme can provide session key security because an adversary cannot compute $M_{TS}=h(q_{i}\left|\right|B_{i}||NU_{i})$. However, we demonstrate that an adversary can compute the value $M_{TS}$ in Section 4.1. Therefore, Mohit et al.’s scheme cannot achieve session key security.

## 5. Proposed Protocol

In this section, we propose a secure authentication protocol for WSNs in vehicle communications to resolve the security problems of Mohit et al.’s scheme [23]. Our proposed scheme consists of four phases: system setup, user registration, login and authentication and password change. In our protocol, the system setup phase is equivalent to that of Mohit et al.’s scheme. The details of the other three phases are presented below.

### 5.1. User Registration Phase

**Figure 5:**
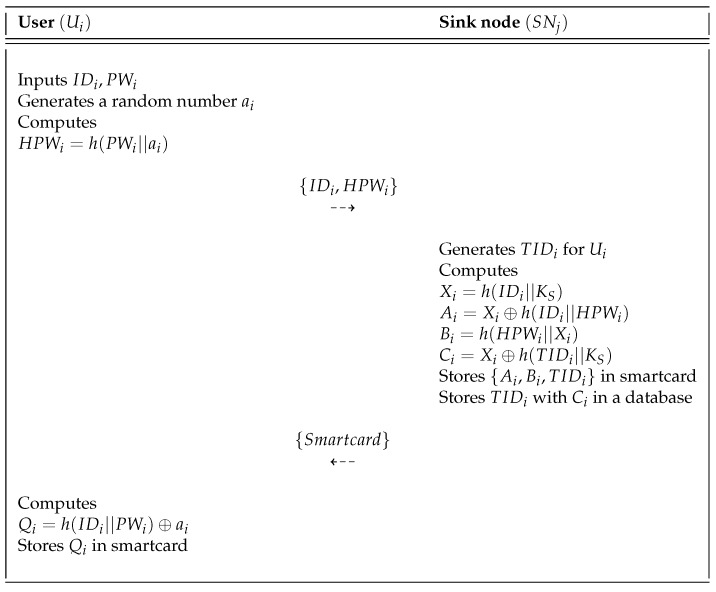
User registration phase of the proposed scheme.

When a new user $U_{i}$ wants to first access the sink node as a traffic manager, he or she must first register with the sink node. The user registration phase of the proposed protocol is shown in Figure 5 and the detailed steps are as follows:

**Step** **1:** The user $U_{i}$ selects the identity $ID_{i}$ and password $PW_{i}$ and then generates a random number $a_{i}$ to computes $HPW_{i}=h(PW_{i}\left|\right|a_{i})$. Then, $U_{i}$ sends $\left\{ID_{i},HPW_{i}\right\}$ to the sink node $SN_{j}$ via a secure channel.
**Step** **2:** After receiving the registration request message from $U_{i}$, $SN_{j}$ generates a random unique identity $TID_{i}$ for $U_{i}$ and computes $X_{i}=h(ID_{i}\left|\right|K_{S}),A_{i}=X_{i}⊕h(ID_{i}||HPW_{i}),B_{i}=h(HPW_{i}\left|\right|X_{i})$, and $C_{i}=X_{i}⊕h(TID_{i}\left|\right|K_{s})$. After that, $SN_{j}$ stores $\left\{A_{i},B_{i},TID_{i}\right\}$ in a smartcard, which it issues to $U_{i}$ through a secure channel. Finally, $SN_{j}$ stores $\left\{TID_{i},C_{i}\right\}$ in a database.
**Step** **3:** Upon receiving the smartcard from $SN_{j}$, $U_{i}$ calculates $Q_{i}=h(ID_{i}||PW_{i})⊕a_{i}$ and stores $\left\{Q_{i}\right\}$ in the smartcard. Consequently, $SN_{j}$ stores $\left\{A_{i},B_{i},TID_{i},Q_{i}\right\}$ in the smartcard.

### 5.2. Login and Authentication Phase

**Figure 6:**
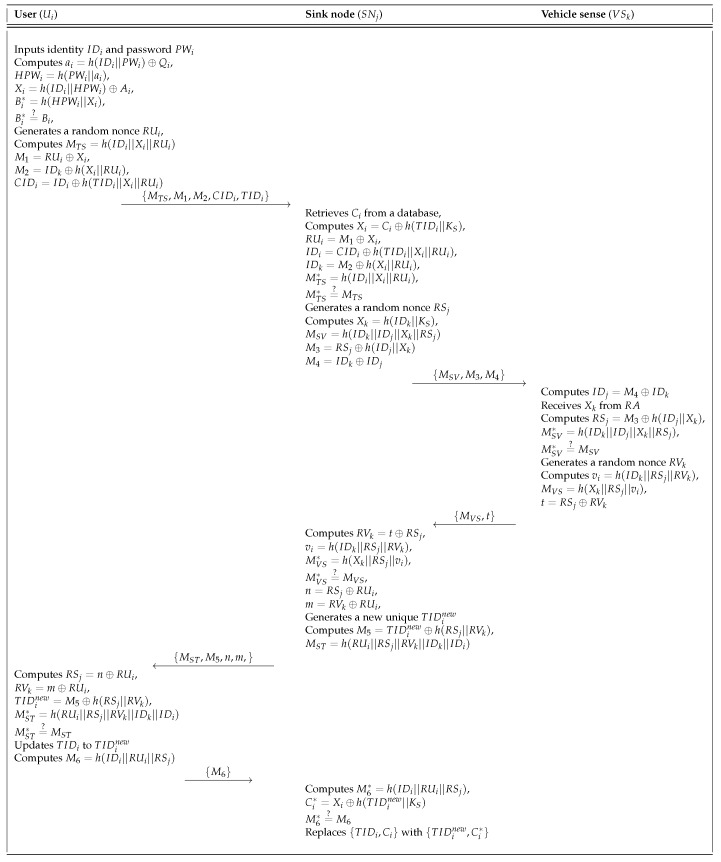
User login and authentication phase of the proposed scheme.

If a user $U_{i}$ wants to access the sink node $SN_{j}$, $U_{i}$ must send a login request message. The login and authentication phase of our scheme is shown in Figure 6 and the details of this phase are as follows.

**Step** **1:** $U_{i}$ inserts the smartcard and inputs the identity $ID_{i}$ and password $PW_{i}$ into a smartcard reader. Then, $U_{i}$ computes $a_{i}=h(ID_{i}||PW_{i})⊕Q_{i},HPW_{i}=h(PW_{i}\left|\right|a_{i}),X_{i}=h(ID_{i}||HPW_{i})⊕A_{i}$, and $B_{i}^{*}=h(HPW_{i}\left|\right|X_{i})$ and checks whether $B_{i}^{*}\overset{?}{=}B_{i}$. If it is equal, $U_{i}$ generates a random nonce $RU_{i}$ and computes $M_{1}=RU_{i}⊕X_{i},M_{2}=ID_{k}⊕h(X_{i}||RU_{i}),CID_{i}=ID_{i}⊕h(TID_{i}\left|\right|X_{i}||RU_{i})$, and $M_{TS}=h(ID_{i}\left|\right|X_{i}||RU_{i})$. $U_{i}$ sends the login request message $\left\{M_{TS},M_{1},M_{2},CID_{i},TID_{i}\right\}$ to $SN_{j}$ through a public channel.
**Step** **2:** After receiving the login request message from $U_{i}$, $SN_{j}$ retrieves $C_{i}$ matched with $TID_{i}$ in a database. Then, $SN_{j}$ computes $X_{i}=C_{i}⊕h(TID_{i}\left|\right|K_{S}),RU_{i}=M_{1}⊕X_{i},ID_{i}=CID_{i}⊕h(TID_{i}\left|\right|X_{i}||RU_{i}),ID_{k}=M_{2}⊕h(X_{i}||RU_{i})$, and $M_{TS}^{*}=h(ID_{i}\left|\right|X_{i}||RU_{i})$ and checks whether $M_{TS}^{*}\overset{?}{=}M_{TS}$. If it is correct, $SN_{j}$ generates a random nonce $RS_{j}$ and computes $X_{k}=h(ID_{k}\left|\right|K_{S}),M_{SV}=h(ID_{k}||ID_{j}\left|\right|X_{k}||RS_{j}),M_{3}=RS_{j}⊕h(ID_{j}\left|\right|X_{k})$, and $M_{4}=ID_{k}⊕ID_{j}$. $SN_{j}$ also sends the authentication request message $\left\{M_{SV},M_{3},M_{4}\right\}$ to $VS_{k}$ via a public channel.
**Step** **3:** Upon receiving the message $\left\{M_{SV},M_{3},M_{4}\right\}$, $VS_{k}$ computes $ID_{j}=M_{4}⊕ID_{k}$ and receives $X_{k}$ from RA. Then, $VS_{k}$ computes $RS_{j}=M_{3}⊕h(ID_{j}\left|\right|X_{k})$ and $M_{SV}^{*}=h(ID_{k}||ID_{j}\left|\right|X_{k}||RS_{j})$ and checks whether $M_{SV}^{*}\overset{?}{=}M_{SV}$. If they are equal, $VS_{k}$ generates a random nonce $RV_{k}$ and computes $v_{i}=h(ID_{k}||RS_{j}||RV_{K}),M_{VS}=h(X_{k}||RS_{j}\left|\right|v_{i})$, and $t=RS_{j}⊕RV_{k}$. Finally, $VS_{k}$ sends $\left\{M_{VS},t\right\}$ to $SN_{j}$ through a public channel.
**Step** **4:** After receiving the message $\left\{M_{VS},t\right\}$ from $VS_{k}$, $SN_{j}$ computes $RV_{k}=t⊕RS_{j},v_{i}=h(ID_{k}||RS_{j}||RV_{k})$ and $M_{VS}^{*}=h(X_{k}||RS_{j}\left|\right|v_{i})$. Then, $SN_{j}$ checks whether $M_{VS}^{*}\overset{?}{=}M_{VS}$. If it is equal, $SN_{j}$ computes $n=RS_{j}⊕RU_{i}$ and $m=RV_{k}⊕RU_{i}$. After that, $SN_{j}$ generates a new random unique identity $TID_{i}^{new}$ and computes $M_{5}=TID_{i}^{new}⊕h(RS_{j}||RV_{k})$ and $M_{ST}=h(RU_{i}||RS_{j}||RV_{k}||ID_{k}||ID_{i})$. $SN_{j}$ also sends the message $\left\{M_{ST},M_{5},n,m\right\}$ to $U_{i}$ via an open channel.
**Step** **5:** Upon receiving the message $\left\{M_{ST},M_{5},n,m\right\}$, $U_{i}$ computes $RS_{j}=n⊕RU_{i},RV_{k}=m⊕RU_{i},TID_{i}^{new}=M_{5}⊕h(RS_{j}||RV_{k})$, and $M_{ST}^{*}=h(RU_{i}||RS_{j}||RV_{k}||ID_{k}||ID_{i})$. Then, $U_{i}$ checks whether $M_{ST}^{*}\overset{?}{=}M_{ST}$. If it is equal, $U_{i}$ updates $TID_{i}$ to $TID_{i}^{new}$. Finally, $U_{i}$ computes $M_{6}=h(ID_{i}||RU_{i}||RS_{j})$ and sends the confirmation message $\left\{M_{6}\right\}$ to $SN_{j}$.
**Step** **6:** After receiving the message $\left\{M_{6}\right\}$ from $U_{i}$, $SN_{j}$ computes $M_{6}^{*}=h(ID_{i}||RU_{i}||RS_{j})$ and $C_{i}^{*}=X_{i}⊕h(TID_{i}^{new}\left|\right|K_{S})$. Then, $SN_{j}$ checks whether $M_{6}^{*}\overset{?}{=}M_{6}$. If it is valid, $SN_{j}$ replaces $\left\{TID_{i},C_{i}\right\}$ with $\left\{TID_{i}^{new},C_{i}^{*}\right\}$.

### 5.3. Password Change Phase

**Figure 7:**
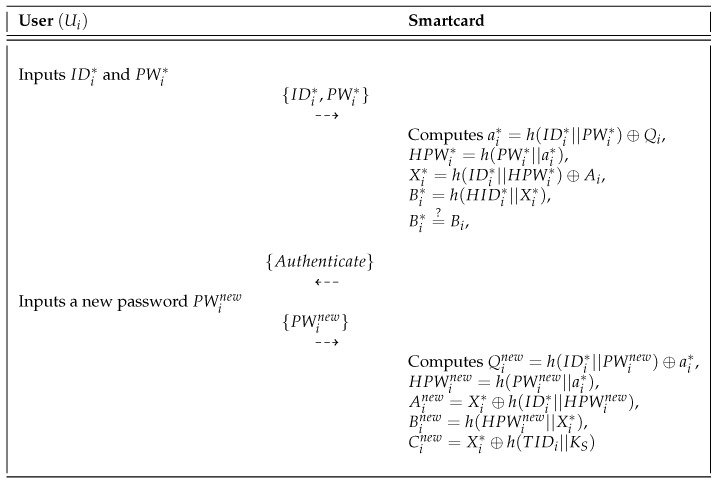
Password change phase of the proposed scheme.

In our proposed protocol, $U_{i}$ can change the password when desired without the help of the sink node $SN_{j}$. The password change phase is shown in Figure 7 and the detailed steps of this phase are presented below:

**Step** **1:** $U_{i}$ inserts his or her smartcard into a card reader and inputs the identity $ID_{i}$ and old password $PW_{i}^{*}$.
**Step** **2:** SC computes $a_{i}^{*}=h(ID_{i}^{*}||PW_{i}^{*})⊕Q_{i},HPW_{i}^{*}=h(PW_{i}^{*}\left|\right|a_{i}^{*}),X_{i}^{*}=h(ID_{i}^{*}||HPW_{i}^{*})⊕A_{i}$, and $B_{i}^{*}=h(HPW_{i}^{*}\left|\right|X_{i}^{*})$. Then, SC compares the computed $B_{i}^{*}$ with the stored $B_{i}$ in its memory. If it is valid, SC sends an authentication message to $U_{i}$.
**Step** **3:** On receiving the message from the smartcard, $U_{i}$ inserts the new password $PW_{i}^{new}$ in the smartcard.
**Step** **4:** Using the new password $PW_{i}^{new}$, SC computes $Q_{i}^{new}=h(ID_{i}^{*}||PW_{i}^{new})⊕a_{i}^{*},HPW_{i}^{new}=h(PW_{i}^{new}\left|\right|a_{i}^{*}),A_{i}^{new}=X_{i}^{*}⊕h(ID_{i}^{*}||HPW_{i}^{new}),B_{i}^{new}=h(HPW_{i}^{new}\left|\right|X_{i}^{*})$, and $C_{i}^{new}=X_{i}^{*}⊕h(TID_{i}\left|\right|K_{s})$. Finally, the smartcard replaces the old information with $\left\{A_{i}^{new},B_{i}^{new},C_{i}^{new},Q_{i}^{new}\right\}$.

## 6. Security Analysis

In this section, we use the Burrow–Abadi–Needham (BAN) logic [28], which is a broadly accepted formal security model, to carry out an analysis and prove that our protocol can provide secure mutual authentication. We also demonstrate that our proposed protocol can resist various attacks through an informal security analysis, which is based on Section 1.1.

### 6.1. Informal Security Analysis

We present an informal security analysis of our proposed scheme to show that it prevents trace, impersonation, and replay attacks. In addition, we demonstrate that our protocol can achieve mutual authentication and anonymity.

#### 6.1.1. Impersonation Attack

If an adversary $U_{a}$ tries to impersonate a legitimate user $U_{i}$, $U_{a}$ must generate a login request message $\left\{M_{TS},M_{1},M_{2},CID_{i},TID_{i}\right\}$ and response message $\left\{M_{6}\right\}$ successfully. However, $U_{a}$ cannot generate these because $U_{a}$ cannot know the real identity of $U_{i}$ and secret parameters $X_{i},RU_{i}$, and $K_{S}$. In addition, $U_{a}$ does not retrieve a random nonce $RU_{i}$ from $M_{1}$. Therefore, our protocol resists impersonation attacks because $U_{a}$ cannot generate valid messages.

#### 6.1.2. Trace Attack and Anonymity

In the login and authentication phase of our protocol, an adversary $U_{a}$ cannot trace a legitimate user $U_{i}$ or vehicle $VS_{k}$ because all transmitted messages are changed every session. In addition, $U_{i}$ sends the dynamic identity $CID_{i}=ID_{i}⊕h(TID_{i}\left|\right|X_{i}||RU_{i})$ and $TID_{i}$ to the sink node, and the identity of $VS_{k}$ is also included in $M_{4}=ID_{k}⊕ID_{j}$. In other words, to obtain the record of a user’s movement and real identity, an adversary must know the user’s real identity $ID_{i}$, secret parameter $X_{i}$, and random nonces $RU_{i}$, $RS_{j}$, and $RV_{k}$. For these reasons, our protocol provides the anonymity and is secure against trace attacks.

#### 6.1.3. Smartcard Stolen Attack

According to Section 1.1, we assume that an adversary $U_{a}$ can obtain a smartcard and extract the parameters $\left\{A_{i},B_{i},TID_{i},Q_{i}\right\}$. However, $U_{a}$ cannot obtain any sensitive user information without $ID_{i}$ and $PW_{i}$ because the parameters stored in smartcards are masked in $X_{i}=h(ID_{i}\left|\right|K_{S})$, $A_{i}=X_{i}⊕h(ID_{i}||HPW_{i})$, $B_{i}=h(HPW_{i}\left|\right|X_{i})$, $C_{i}=X_{i}⊕h(TID_{i}\left|\right|K_{S})$, and $Q_{i}=h(ID_{i}||PW_{i})⊕a_{i}$ by the hash function and XOR operation. Consequently, our proposed protocol prevents smartcard stolen attack.

#### 6.1.4. Replay Attack

According to Section 1.1, we suppose that adversary $U_{a}$ tries to impersonate a legitimate user $U_{i}$ by resending messages transmitted in the previous session, $U_{a}$ cannot impersonate $U_{i}$ successfully. In our scheme, the sink node $SN_{j}$ checks whether a random nonce is fresh or not. If a random nonce value $RU_{i}$ is not fresh, $SN_{j}$ rejects the login request message. In addition, $U_{a}$ cannot generate the confirmation message $M_{6}$ successfully because $U_{a}$ cannot obtain the random nonce $RS_{j}$ generated by $SN_{j}$. Therefore, the proposed protocol is secure against replay attacks.

#### 6.1.5. Secure Mutual Authentication

When receiving the login message $\left\{M_{TS},M_{1},M_{2},CID_{i},TID_{i}\right\}$ and confirmation message $\left\{M_{6}\right\}$ from $U_{i}$, the sink node $SN_{j}$ checks whether $M_{TS}$ and $M_{6}$ are correct. In addition, $SN_{j}$ retrieves $X_{i}$ from a database to validate $M_{TS}$. If this is correct, $SN_{j}$ authenticates $U_{i}$. After receiving $\left\{M_{VS},t\right\}$ from $VS_{k}$, the sink node checks whether $M_{SV}=h(ID_{k}||RS_{j}||RV_{k})$ is valid. If it is valid, $SN_{j}$ authenticates $VS_{k}$. Finally, the user $U_{i}$ checks whether the received value $M_{ST}=h(RU_{i}||RS_{j}||RV_{k}||ID_{k}||ID_{i})$ is correct. If it is correct, $U_{i}$ authenticates $SN_{j}$. Therefore, all entities authenticate each other successfully because an adversary cannot know the important parameters discussed in Section 6.1.1 and Section 6.1.2.

According to Section 6.1.2 and Section 6.1.5, all transmitted messages are changed every session and an adversary cannot obtain user’s sensitive information. Therefore, we achieve essential security requirement into untraceability, anonymity, secure mutual authentication and confidentiality. Furthermore, secure mutual authentication is proved in Section 6.2 using BAN logic.

### 6.2. Security Analysis Using BAN Logic

To prove the secure mutual authentication of our protocol, we perform an analysis with the BAN logic [28], which is a widely accepted formal security model. First, we define the notation of the BAN logic in Table 2. Then, we describe the logical postulates of the BAN logic in Section 6.2.1. Next, we present the goals, idealized form, and initial assumptions of our protocol. Finally, we demonstrate that our protocol achieves secure mutual authentication between $U_{i}$ and $VK_{k}$ by using the BAN logic.

#### 6.2.1. Postulates of BAN Logic

The postulates of the BAN logic are given below:

**1.**  Message meaning rule :
  $$\frac{P\;|\equiv P\overset{K}{↔}Q,\;P⊲{\left\{X\right\}}_{K}}{P\;\left|\equiv Q\;\right|∼X},$$
**2.**  Nonce verification rule :
  $$\frac{P\;\left|\equiv \#(X),\;P\;\right|\equiv Q\;|∼X}{P\;\left|\equiv Q\;\right|\equiv X},$$
**3.**  Jurisdiction rule :
  $$\frac{P\;\left|\equiv Q\;\right|⟹X,\;P\;\left|\equiv Q\;\right|\equiv X}{P\;|\equiv X},$$
**4.**  Freshness rule :
  $$\frac{P\;|\equiv \#(X)}{P\;|\equiv \#\left(X,Y\right)},$$
**5.**  Belief rule :
  $$\frac{P\;|\equiv \left(X,Y\right)}{P\;|\equiv X}.$$

#### 6.2.2. Goals

We have the following goals to prove the secure mutual authentication of our proposed protocol:

**Goal** **1:** $U_{i}|\equiv \left(RS_{j},RV_{k}\right),$
**Goal** **2:** $U_{i}|\equiv SN_{j}|\equiv \left(RS_{j},RV_{k}\right),$
**Goal** **3:** $SN_{j}|\equiv \left(RU_{i}\right),$
**Goal** **4:** $SN_{j}|\equiv U_{i}|\equiv \left(RU_{i}\right),$
**Goal** **5:** $SN_{j}|\equiv \left(RV_{k}\right),$
**Goal** **6:** $SN_{j}|\equiv VS_{k}|\equiv \left(RV_{k}\right).$

#### 6.2.3. Idealized Forms

The idealized forms of the transmitted messages are given below:

*Msg*_1_: $U_{i}\to SN_{j}$: ${\left(ID_{i},ID_{k},TID_{i},RU_{i}\right)}_{X_{i}},$
*Msg*_2_: $SN_{j}\to VS_{k}$: ${\left(ID_{i},ID_{k},RU_{i}\right)}_{X_{k}},$
*Msg*_3_: $VS_{k}\to SN_{j}$: ${\left(ID_{k},RS_{j},RV_{k}\right)}_{X_{k}},$
*Msg*_4_: $SN_{j}\to U_{i}$: $({ID_{k},TID_{i}^{new},RU_{i},RS_{j},RV_{k})}_{ID_{i}},$
*Msg*_5_: $U_{i}\to SN_{j}$: $({RU_{i},RS_{j})}_{ID_{i}}.$

#### 6.2.4. Assumptions

We make the following initial assumptions to perform the BAN logic proof:

*A*_1_: $U_{i}|\equiv \left(U_{i}\overset{X_{i}}{⟷}SN_{j}\right),$
*A*_2_: $SN_{j}|\equiv \left(U_{i}\overset{X_{i}}{⟷}SN_{j}\right),$
*A*_3_: $VS_{k}|\equiv \left(VS_{k}\overset{X_{k}}{⟷}SN_{j}\right),$
*A*_4_: $SN_{j}|\equiv \left(VS_{k}\overset{X_{k}}{⟷}SN_{j}\right),$
*A*_5_: $SN_{j}|\equiv \#\left(RU_{i}\right),$
*A*_6_: $VS_{k}|\equiv \#\left(RS_{j}\right),$
*A*_7_: $SN_{j}|\equiv \#\left(RV_{k}\right),$
*A*_8_: $U_{i}|\equiv \#\left(RS_{j}\right),$
*A*_9_: $U_{i}|\equiv \left(U_{i}\overset{ID_{i}}{⟷}SN_{j}\right),$
*A*_10_: $U_{i}|\equiv SN_{j}\Rightarrow \left(RS_{j},RV_{k}\right),$
*A*_11_: $SN_{j}|\equiv U_{i}\Rightarrow \left(RU_{i}\right),$
*A*_12_: $SN_{j}|\equiv VS_{k}\Rightarrow \left(RV_{k}\right).$

#### 6.2.5. Proof Using BAN Logic

The detailed steps of the main proof are as follows:

**Step** **1:** According to $Msg_{1}$, we can obtain
  $$S_{1}:SN_{j}⊲{\left(ID_{i},ID_{k},TID_{i},RU_{i}\right)}_{X_{i}}.$$
**Step** **2:** In conformity with the message meaning rule with $S_{1}$ and $A_{2}$, we can get
  $$S_{2}:SN_{j}|\equiv U_{i}∼{\left(ID_{i},ID_{k},TID_{i},RU_{i}\right)}_{X_{i}}.$$
**Step** **3:** According to the freshness rule with $A_{5}$, we can get
  $$S_{3}:SN_{j}|\equiv \#{\left(ID_{i},ID_{k},TID_{i},RU_{i}\right)}_{X_{i}}.$$
**Step** **4:** According to the nonce verification rule with $S_{2}$ and $S_{3}$, we can obtain
  $$S_{4}:SN_{j}|\equiv U_{i}|\equiv {\left(ID_{i},ID_{k},TID_{i},RU_{i}\right)}_{X_{i}}.$$
**Step** **5:** According to $Msg_{2}$, we can get
  $$S_{5}:VS_{k}⊲{\left(ID_{i},ID_{k},RU_{i}\right)}_{X_{k}}.$$
**Step** **6:** In conformity with the message meaning rule with $S_{5}$ and $A_{3}$, we can get
  $$S_{6}:VS_{k}|\equiv SN_{j}∼{\left(ID_{i},ID_{k},RU_{i}\right)}_{X_{k}}.$$
**Step** **7:** According to the freshness rule with $A_{6}$, we can obtain
  $$S_{7}:VS_{k}|\equiv \#{\left(ID_{i},ID_{k},RU_{i}\right)}_{X_{k}}.$$
**Step** **8:** According to the nonce verification rule with $S_{6}$ and $S_{7}$, we can get
  $$S_{8}:VS_{k}|\equiv SN_{j}|\equiv {\left(ID_{i},ID_{k},RU_{i}\right)}_{X_{k}}.$$
**Step** **9:** According to $Msg_{3}$, we can obtain
  $$S_{9}:SN_{j}⊲{\left(ID_{k},RS_{j},RV_{k}\right)}_{X_{k}}.$$
**Step** **10:** In conformity with the message meaning rule with $S_{9}$ and $A_{4}$, we can obtain
  $$S_{10}:SN_{j}|\equiv VS_{k}∼{\left(ID_{k},RS_{j},RV_{k}\right)}_{X_{k}}.$$
**Step** **11:** According to the freshness rule with $A_{7}$, we can get
  $$S_{11}:SN_{j}|\equiv \#{\left(ID_{k},RS_{j},RV_{k}\right)}_{X_{k}}.$$
**Step** **12:** According to the nonce verification rule with $S_{10}$ and $S_{11}$, we can get
  $$S_{12}:SN_{j}|\equiv VS_{k}|\equiv {\left(ID_{k},RS_{j},RV_{k}\right)}_{X_{k}}.$$
**Step** **13:** According to $Msg_{4}$, we can obtain
  $$S_{13}:U_{i}⊲({ID_{k},TID_{i}^{new},RU_{i},RS_{j},RV_{k})}_{ID_{i}}.$$
**Step** **14:** In conformity with the message meaning rule with $S_{13}$ and $A_{9}$, we can get
  $$S_{14}:U_{i}|\equiv SN_{j}∼({ID_{k},TID_{i}^{new},RU_{i},RS_{j},RV_{k})}_{ID_{i}}.$$
**Step** **15:** According to the freshness rule with $A_{8}$, we can get
  $$S_{15}:U_{i}|\equiv \#({ID_{k},TID_{i}^{new},RU_{i},RS_{j},RV_{k})}_{ID_{i}}.$$
**Step** **16:** According to the nonce verification rule with $S_{14}$ and $S_{15}$, we can get
  $$S_{16}:ID_{i}|\equiv SN_{j}|\equiv ({ID_{k},TID_{i}^{new},RU_{i},RS_{j},RV_{k})}_{ID_{i}}.$$
**Step** **17:** According to the belief rule with $S_{16}$, we can get
  $$S_{17}:U_{i}|\equiv SN_{j}|\equiv \left(RS_{j},RV_{k}\right).\;\;\left(\mathrm{Goal}2\right)$$
**Step** **18:** In conformity with the jurisdiction rule with $S_{17}$ and $A_{10}$, we can obtain
  $$S_{18}:U_{i}|\equiv \left(RS_{j},RV_{k}\right).\;\;\left(\mathrm{Goal}1\right)$$
**Step** **19:** In conformity with the belief rule with $S_{4}$, we can get
  $$S_{19}:SN_{j}|\equiv U_{i}|\equiv \left(RU_{i}\right).\;\;\left(\mathrm{Goal}4\right)$$
**Step** **20:** According the jurisdiction rule with $S_{19}$ and $A_{11}$, we can obtain
  $$S_{20}:SN_{j}|\equiv \left(RU_{i}\right).\;\;\left(\mathrm{Goal}3\right)$$
**Step** **21:** In conformity with the belief rule with $S_{12}$, we can get
  $$S_{21}:SN_{j}|\equiv VS_{k}|\equiv \left(RV_{k}\right).\;\;\left(\mathrm{Goal}6\right)$$
**Step** **22:** According the jurisdiction rule with $S_{19}$ and $A_{11}$, we can obtain
  $$S_{20}:SN_{j}|\equiv \left(RV_{k}\right).\;\;\left(\mathrm{Goal}5\right)$$

Based on goals 1–6, we prove that our proposed protocol achieves secure mutual authentication between $U_{i}$ and $VS_{k}$.

## 7. Security Analysis Using the AVISPA Tool

In this section, we perform a formal security verification of our protocol with the widely accepted Automated Validation of Internet Security Protocols and Applications (AVISPA) simulation tool [33,34]. Formal security verification with this tool has received much attention and has been used in numerous studies to demonstrate that various authentication protocols are secure against replay and man-in-the-middle attacks [35,36,37,38,39].

With AVISPA, the security protocol must be implemented by using the High Level Protocols Specification Language (HLPSL) [40]. The HLPSL specifications of the security protocol are translated to an intermediate format (IF) by the HLPSLIF translator. Finally, it is converted to the output format (OF) with the On-the-fly Model-Checker (OFMC) [41], the CL-based Attack Searcher (AtSe) [42], SAT-based Model-Checker (SATMC), or Tree Automata-based Protocol Analyzer (TA4SP).

### 7.1. HLPSL Specifications

**Figure 8:**
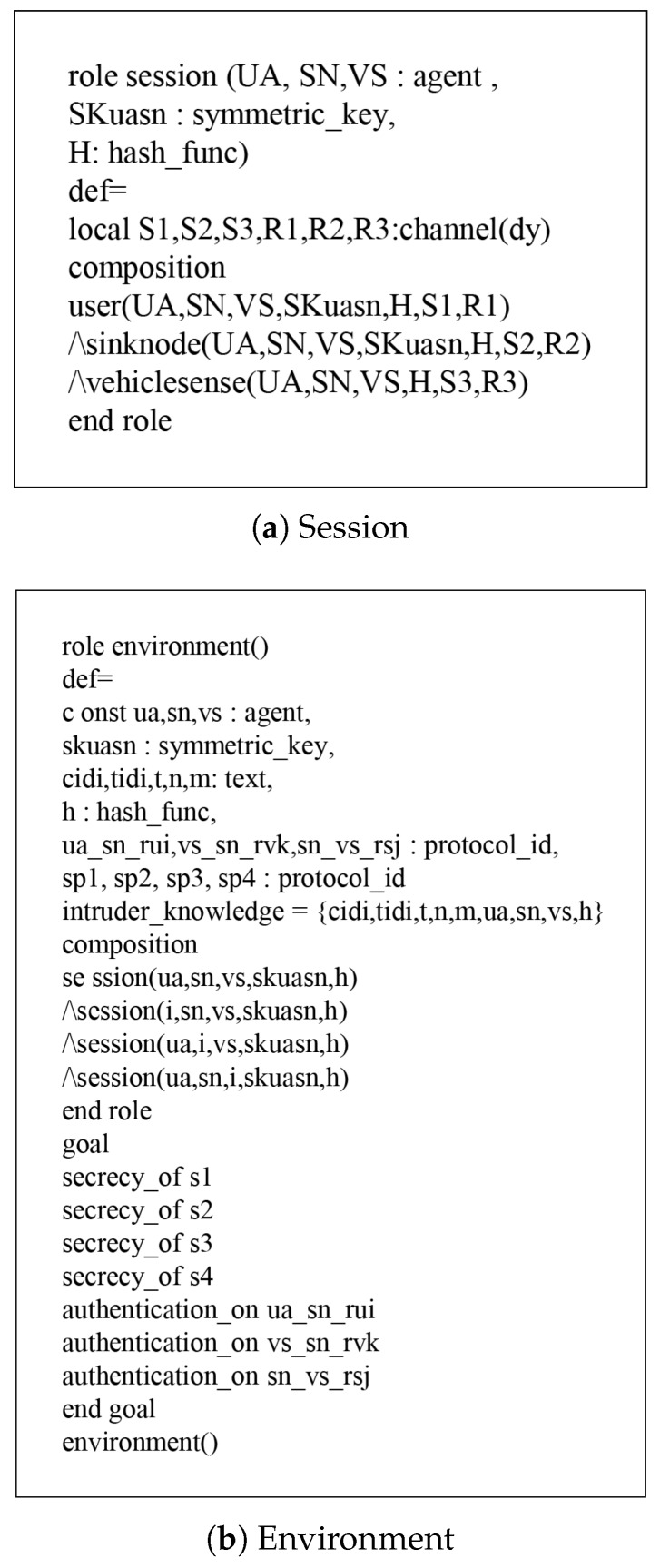
Role specification for session and environment.

**Figure 9:**
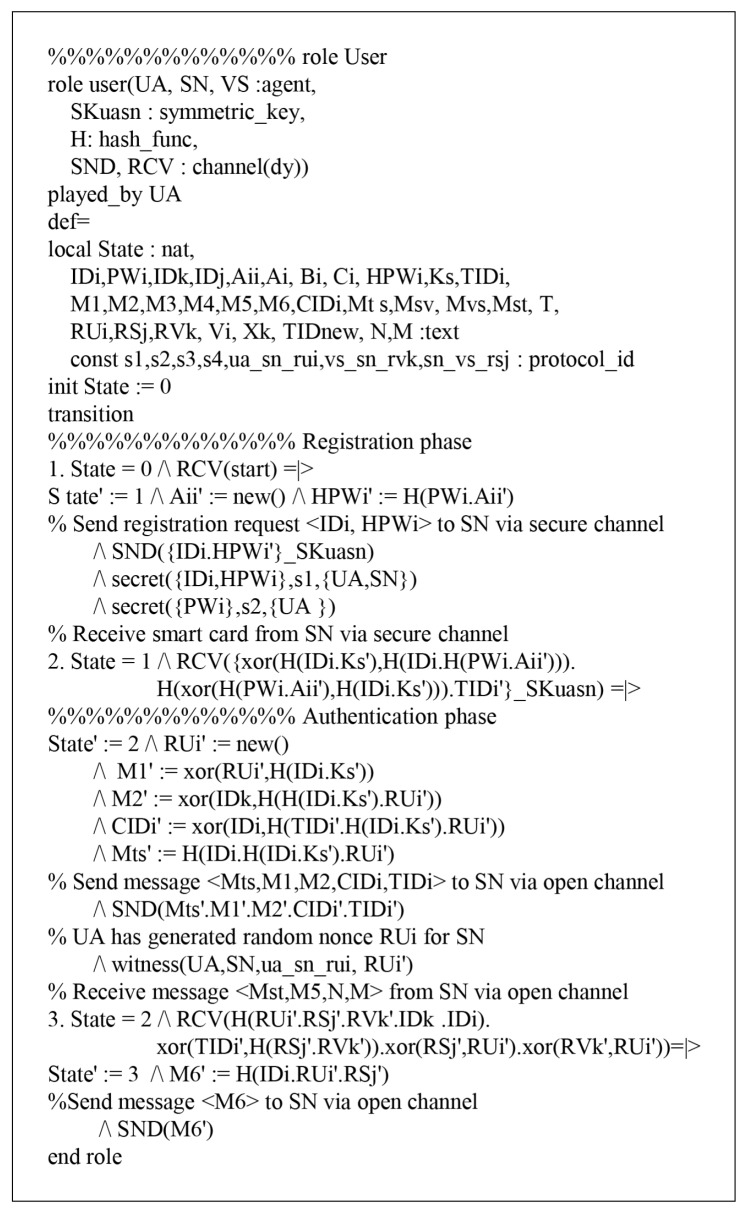
Role specification for user UA.

According to HLPSL, the proposed protocol has three entities, which are called role: user denotes a user UA, sinknode denotes a sink node SN, and vehiclesense denotes a vehicle sense VS. The session and environment also contain the security goals, as shown in Figure 8. The role specifications of $U_{i}$ are shown in Figure 9 and the details are as follows.

**Figure 10:**
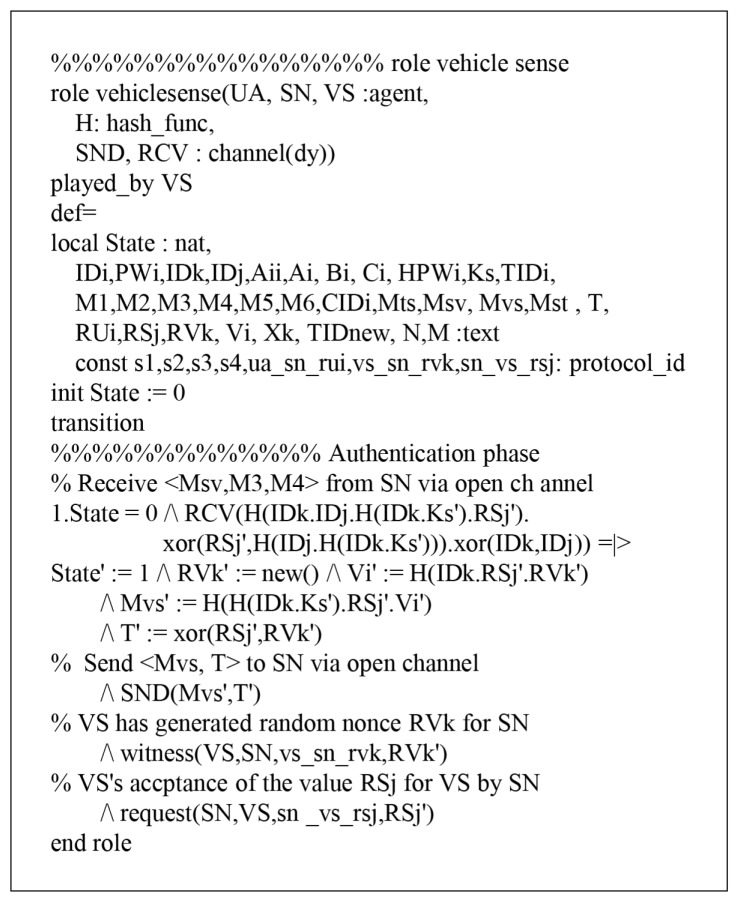
Role specification for VS.

**Figure 11:**
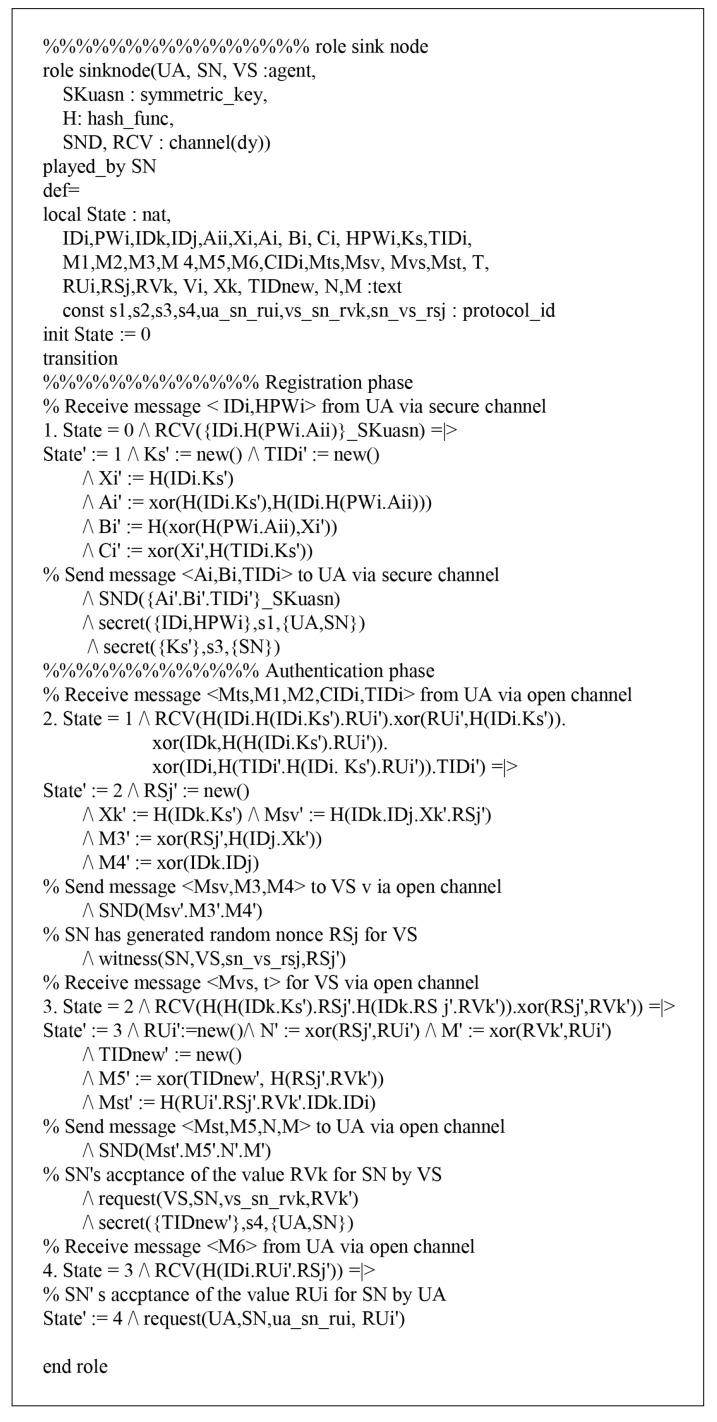
Role specification for SN.

When $U_{i}$ receives the start message, UA changes the state value 0 to 1. Then, UA sends the registration request $\left\{ID_{i},HPW_{i}\right\}$ to SN via a secure channel and receives the smartcard from SN. After that, UA updates the state from 1 to 2. During the login and authentication phase, UA sends the login message $\left\{M_{ts},M_{1},M_{2},CID_{i},TID_{i}\right\}$ to SN via a public channel. Then, UA declares $witness(UA,SN,ua\_sn\_rui,RU_{i}^{\prime })$, which means that it generates a random nonce $RU_{i}$. After generating $RU_{i}$, UA receives the message $\left\{M_{st},M_{5},n,m\right\}$ from SN and updates the state from 2 to 3. Finally, UA sends $\left\{M_{6}\right\}$ to SN through a public channel and SN authenticates UA by using a random nonce $RU_{i}$. Similarly, the simulated results of SN and VS are defined as shown in Figure 10 and Figure 11.

### 7.2. Analysis of Simulation Results

**Figure 12:**
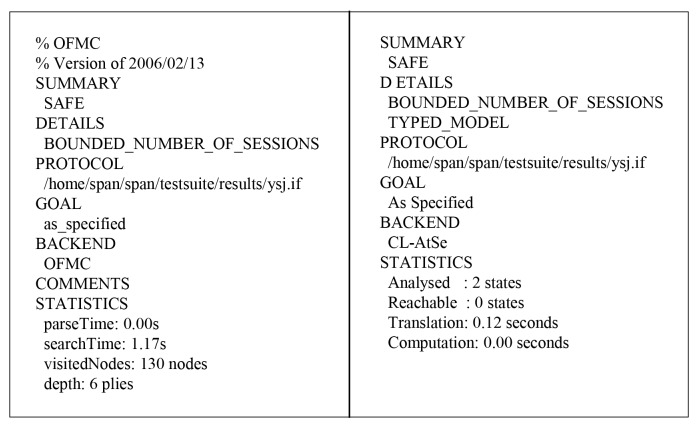
The result of analysis using OFMC and CL-AtSe

In this section, we present the results of the AVISPA analysis using OFMC and CL-AtSe back-ends to ensure the security of our protocol, as shown in Figure 12. To estimate the security against replay attack, the OFMC and CL-AtSe back-ends check whether a legitimate entity can execute the protocol by searching for a passive adversary. Moreover, the OFMC and CL-AtSe back-ends also check whether the proposed protocol is secure against the man-in-the-middle attack for the DY model checking.

The OFMC back-end has a search time of 1.17 seconds to visit 130 nodes, and the CL-AtSe back-end analyzes two states with a translation time of 0.12 seconds. Because the replay attack and Dolev–Yao model checking are performed successfully, the proposed protocol is safe against replay and man-in-the-middle attacks.

## 8. Performance Analysis

In this section, we compare the computation and communication costs of our proposed protocol with those of related protocols [3,15,16,23,43,44] and discuss the security properties.

### 8.1. Computation Cost

We compare the computation overheads of our protocol with those of related protocols [3,15,16,23,43,44]. For the comparison of computation cost, we define the notations as follows. $T_{h}$, $T_{S}$, and $T_{M}$ denote the times for hash operation (≈0.0005 s), symmetric key cryptographic operation (≈0.0087 s) and elliptic curve scalar point multiplication operation (≈0.0630 s), respectively. The analysis results are presented in Table 3.

We use the existing computation analysis results of Mohit et al. [23] for a rough evaluation. We do not include the XOR operation because it is negligible compared with the other operations. The results show that our protocol needs $8T_{h}$ for the user, $13T_{h}$ for the sink node, and $4T_{h}$ for the sensor. Thus, total cost of our protocol is 0.0125 seconds. Even though this is slightly higher than the cost for Mohit et al.’s protocol, the difference is negligible, and the proposed protocol provides better security than other protocols. Therefore, our protocol is secure and suitable for practical WSNs environments.

### 8.2. Security Properties

Table 4 compares the security properties of our proposed protocol compared with other related protocols. The existing related schemes clearly cannot resist various attacks, and their protocols cannot achieve anonymity and mutual authentication. For these reasons, our protocol provides better security features than the other protocols [3,15,16,23,43,44].

### 8.3. Communication Cost

Finally, we analyze the communication cost of our scheme with related protocols. For the communication analysis, we assume that a random nonce (number) and timestamp are 64 bits, a pseudo-identity is 160 bits, the SHA-1 hash digest [45] is 160 bits, elliptic curve scalar multiplication is 512 bits, and symmetric key cryptographic operation is 256 bits. In the login and authentication phase of our protocol, the transmitted messages $\left\{M_{TS},M_{1},M_{2},CID_{i},TID_{i}\right\},\left\{M_{SV},M_{3},M_{4}\right\},\left\{M_{VS},t\right\},\left\{M_{ST},M_{5},n,m,\right\},and\left\{M_{6}\right\}$ require (160+64+64+160+160=608 bits), (160+64+64=288 bits), (160+64=224 bits), (160+160+64+64=448 bits) and 160 bits, respectively. Consequently, the total communication cost is (608+288+224+448+160=1728 bits). Table 5 presents the results of this analysis. Even though our protocol has a higher communication cost than Mohit et al.’s scheme, the vehicle sense sends only 224 bits, which is similar to that of their scheme. Therefore, from the perspective of limited resources, the proposed scheme is sufficiently applicable to WSN environments.

## 9. Conclusions

In this paper, we demonstrate that Mohit et al.’s scheme does not resist the impersonation and trace attacks. We also show that it does not achieve secure mutual authentication, session key security, and anonymity. We propose a secure authentication protocol for WSNs in vehicular communications to resolve the security problems of their scheme. The proposed protocol is secure against impersonation, replay, smartcard stolen and trace attacks and can achieve secure mutual authentication and anonymity by using dynamic values for the transmitted messages that change every session. We also prove that our protocol can provide secure mutual authentication between $U_{i}$, $SN_{j}$ and $VS_{k}$ by using BAN logic and we present a formal security verification using the AVISPA tool. Furthermore, we compare the performance and security functionalities with those of other related protocols. Therefore, the proposed protocol can be efficiently applied to practical vehicle communications systems.

## Figures and Tables

**Table 1:** Notations.

Notation	Description
$ID_{i}$	Identity of user
$ID_{j}$	Identity of sink node
$ID_{k}$	Identity of vehicle sensor
$PW_{i}$	Password of user
RA	Registration authority
$a_{i}$	Random number by user
$RU_{i}$	Random nonce by user
$RS_{j}$	Random nonce by sink node
$RV_{k}$	Random nonce by vehicle sensor
$K_{S}$	Master key of sink node
$TID_{i}$	Unique temporary identity of user
h(·)	One-way hash function
⊕	Bitwise XOR operation
||	Concatenation operation

**Table 2:** Notations of the BAN logic.

Notation	Description
P|≡X	*P***believes** the statement *X*
#X	The statement *X* is **fresh**
P⊲X	*P***sees** the statement *X*
P|∼X	*P* once **said** *X*
P⇒X	*P***controls** the statement *X*
$<X{>}_{Y}$	Formula *X* is **combined** with the formula *Y*
${\left\{X\right\}}_{K}$	Formula *X* is **encrypted** by the key *K*
$P\overset{K}{↔}Q$	*P* and *Q* communicate using *K* as the **shared key**
SK	Session key used in the current authentication session

**Table 3:** Computation cost of our proposed scheme with other related schemes.

Schemes	User	Sink Node	Sensor	Total Cost	Total Cost (s)
Shi et al. [15]	$5T_{h}+3T_{M}$	$3T_{h}+2T_{M}$	$4T_{h}+T_{M}$	$12T_{h}+6T_{M}$	0.3840
Choi et al. [16]	$12T_{h}+3T_{M}$	$5T_{h}+T_{M}$	$7T_{h}+2T_{M}$	$24T_{h}+6T_{M}$	0.3900
He et al. [43]	$4T_{h}+2T_{s}$	$2T_{h}+5T_{s}$	$T_{h}+2T_{s}$	$7T_{h}+9T_{s}$	0.0818
Xue et al. [44]	$10T_{h}$	$14T_{h}$	$6T_{h}$	$30T_{h}$	0.0150
Kumari and Om [3]	$10T_{h}$	$8T_{h}$	$6T_{h}$	$24T_{h}$	0.0120
Mohit et al. [23]	$7T_{h}$	$9T_{h}$	$4T_{h}$	$20T_{h}$	0.0100
Ours	$8T_{h}$	$13T_{h}$	$4T_{h}$	$25T_{h}$	0.0125

$T_{h}$: One-way hash operation, $T_{s}$: Symmetric key cryptographic operation, $T_{M}$: Elliptic curve scalar point multiplication operation.

**Table 4:** Security properties of our proposed scheme with other related schemes.

Security Property	Shi et al. [15]	Choi et al. [16]	He et al. [43]	Xue et al. [44]	Kumari and Om [3]	Mohit et al. [23]	Ours
Impersonation attack	∘	∘	∘	∘	×	×	∘
Smartcard stolen attack	×	∘	∘	∘	∘	×	∘
Password change attack	∘	×	×	×	∘	∘	∘
Replay attack	∘	∘	∘	∘	∘	∘	∘
Trace attack	×	×	×	×	×	×	∘
Anonymity	×	×	∘	×	×	×	∘
Mutual authentication	∘	∘	∘	∘	×	×	∘

∘: preserves the security properties, ×: does not preserve the security properties.

**Table 5:** Communication cost of our proposed scheme with other related schemes.

Schemes	Communication Cost
Shi et al. [15]	3968 bits
Choi et al. [16]	3584 bits
He et al. [43]	1216 bits
Xue et al. [44]	1920 bits
Kumari and Om [3]	2048 bits
Mohit et al. [23]	1280 bits
Ours	1728 bits
